# Consistent Reanalysis of Genome-wide Imprinting Studies in Plants Using Generalized Linear Models Increases Concordance across Datasets

**DOI:** 10.1038/s41598-018-36768-4

**Published:** 2019-02-04

**Authors:** Stefan Wyder, Michael T. Raissig, Ueli Grossniklaus

**Affiliations:** 1 Department of Plant and Microbial Biology & Zurich-Basel Plant Science Center, University of Zurich, Zollikerstrasse 107, CH-8008 Zurich, Switzerland; 2 Present Address: Centre for Organismal Studies, Heidelberg University, Im Neuenheimer Feld 230, 69120 Heidelberg, Germany

## Abstract

Genomic imprinting leads to different expression levels of maternally and paternally derived alleles. Over the last years, major progress has been made in identifying novel imprinted candidate genes in plants, owing to affordable next-generation sequencing technologies. However, reports on sequencing the transcriptome of hybrid F1 seed tissues strongly disagree about how many and which genes are imprinted. This raises questions about the relative impact of biological, environmental, technical, and analytic differences or biases. Here, we adopt a statistical approach, frequently used in RNA-seq data analysis, which properly models count overdispersion and considers replicate information of reciprocal crosses. We show that our statistical pipeline outperforms other methods in identifying imprinted genes in simulated and real data. Accordingly, reanalysis of genome-wide imprinting studies in *Arabidopsis* and maize shows that, at least for *Arabidopsis*, an increased agreement across datasets could be observed. For maize, however, consistent reanalysis did not yield a larger overlap between the datasets. This suggests that the discrepancy across publications might be partially due to different analysis pipelines but that technical, biological, and environmental factors underlie much of the discrepancy between datasets. Finally, we show that the set of genes that can be characterized regarding allelic bias by all studies with minimal confidence is small (~8,000/27,416 genes for *Arabidopsis* and ~12,000/39,469 for maize). In conclusion, we propose to use biologically replicated reciprocal crosses, high sequence coverage, and a generalized linear model approach to identify differentially expressed alleles in developing seeds.

## Introduction

In a diploid cell, the maternal and paternal alleles of a given gene usually share the same expression state in a specific tissue, meaning that they are either both expressed or both silent. Important exceptions to this rule are genes regulated by genomic imprinting, where the expression state depends on the parental origin of the alleles, and only one is expressed while the other remains silent or is weakly expressed. The two alleles do not differ in their sequence but rather carry parent-specific, epigenetic imprints that allow the cell to distinguish the two alleles^1–8^. Genomic imprinting evolved independently in mammals and flowering plants (angiosperms) (reviewed in^9–15^). In both groups, offspring develop within the mother and depend solely on her to supply nutrients for growth and development. This common reproductive strategy results in an intragenomic parental conflict over resource allocation, which likely underlies the evolution of genomic imprinting, at least for loci that control growth^14,16,17^. Accordingly, some imprinted genes in both, mammals and plants, have a role in controlling growth (e.g.^18–26^). Consistent with this function, many imprinted genes are preferentially expressed in the tissues that support embryonic growth, i.e. the placenta in mammals or the triploid endosperm in the seeds of flowering plants.

Over the last decade, the advent of Next-Generation Sequencing (NGS) allowed (nearly) genome-wide imprinting studies by sequencing the transcriptome of hybrid F1 seed tissues: Given exonic polymorphisms between the parents, reads overlapping heterozygous SNPs can be assigned to their parent-of-origin, and reciprocal crosses allow the discrimination between parent-of-origin-dependent and strain-specific genetic effects. Accordingly, a number of research groups performed genome-wide, allele-specific transcriptome profiling studies of hybrid seeds in *Arabidopsis* and maize to identify genes that are preferentially expressed from one parental allele^27–38^. As a result, the total number of imprinted genes increased from around 20^6^ to over 900 potentially imprinted plant genes^28–33,35,36,38^.

However, comparisons of the identified imprinted candidate genes revealed little overlap between the studies^30,34,39^. In general, the analysis of RNA-sequencing (RNA-seq) data to identify allele-specific expression is prone to false positives due to both, biological and technical variation^40–42^. Thus, even studies with seemingly similar design heavily disagree on the number of imprinted genes in the mouse brain, e.g. ranging from less than 200^40^ to over a thousand^43^. To date, although guidelines for the analysis of allele-specific expression have recently become available^42^, many different methods have been applied to filter, normalize, and statistically assess allelic imbalance from RNA-seq data. For the analysis of allele-specific expression, several analysis methods and software^42^ have been developed, yet only very few are suitable for an analysis of imprinted expression. Moreover, no specialized method is available for statistical testing of imprinting in the triploid endosperm, where the expected allelic ratio is 2:1 because the mother contributes two genomes to this tissue. In plants, many authors have used count tests (such as Chi-Square, binomial, or Fisher’s exact tests), which heavily underestimate the count dispersion typically observed in RNA-seq data^41,42,44^, resulting in increased numbers of false positives particularly for large counts. Highly expressed transcripts may appear imprinted with high statistical significance, as count tests are sensitive to very small allelic imbalance at high counts, requiring additional filtering with somewhat arbitrary imbalance cut-offs.

Here, we present a new statistical approach to call imprinted genes from large allele-specific RNA-seq datasets from endosperm that outperforms other methods in simulated and real data. We propose a generally applicable approach using generalized linear models (GLM) implemented in edgeR^45^, which is based on the negative binomial distribution to deal with potential count overdispersion^46^ as it is typically seen in RNA-seq data. The presented pipeline outperforms other methods using simulated data. Furthermore, we reanalyze the raw data from seven studies to assess the relative importance of differences in data generation and data analysis. The consistent reanalysis by the proposed pipeline results in a larger overlap of imprinted candidate genes across *Arabidopsis* datasets, but showed little improvement across maize datasets. In conclusion, consistent data analysis can improve concordance between datasets but biological and technical variation in data generation contributes most to the differences among datasets.

## Results

### Comparison of Genome-wide Imprinting Studies in Plants: Biological, Technical, and Statistical Differences

To shed light onto discrepancies between published studies and potential biases, we compared the genome-wide, allele-specific transcriptome profiling studies of hybrid seeds in *Arabidopsis* and maize that were designed to identify imprinted candidate genes in the endosperm. All studies were based on reciprocal crosses of two polymorphic inbred strains, and all studies used manually dissected endosperm. For simplicity, we only compared studies using L*er* and Col-0 accessions (*Arabidopsis*) or B73 and Mo17 inbreds (maize). First, we set out to identify the genes that can be evaluated for imprinting by all studies and reanalyzed the raw data from the seven published studies starting from the raw reads. We examined all reads overlapping with previously known exonic SNPs (see Methods), separated and counted maternal and paternal reads overlapping SNPs, and summed up informative (i.e. SNP containing) reads across transcripts. After discarding very lowly expressed genes (<10 counts per gene), median reads per transcript were 95–654 for the different datasets.

**Figure 1:**
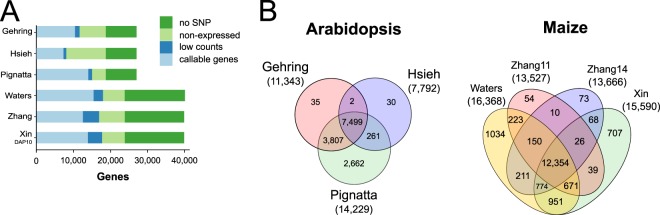
Power of detecting imprinted candidate genes in reanalyzed datasets. (**A**) Callable genes assessable for genomic imprinting were required to overlap with at least 1 exonic SNP and to have read counts of at least 10. Number of genes are shown as percentages of the total number of genes. (**B**) Venn diagrams showing the overlap of callable genes for *Arabidopsis* and maize datasets.

The power of allelic imbalance detection depends mainly on the degree of allelic bias, the sequencing read length and coverage (expression strength), as well as the divergence of the crossed strains (number of SNPs per transcript)^47^. Although the three *Arabidopsis* studies all use the same accessions, the number of callable genes ranges from 7,792 for the Hsieh dataset to 14,229 for the Pignatta dataset (Fig. 1A): Among the 27,416 protein-coding *Arabidopsis* genes, 31% do not overlap with any exonic SNP and their imprinting cannot be assessed. Another 17–39% of the genes are not or only very weakly expressed (<10 allelic counts overall), and were thus discarded from further analysis. Ultimately, only 28–52% of all predicted *Arabidopsis* genes can be assessed for genomic imprinting in the three different studies, because only those have a sufficient number of allele-specific reads to identify statistically significant biased expression (Fig. 1A). 7,499 genes (27% of all *Arabidopsis* genes) can be assessed for genomic imprinting in all three datasets (Fig. 1B).

Among the 39,469 maize protein-coding genes, 33% do not overlap with any exonic SNP (Fig. 1A) between the B73 and Mo17 inbreds. Another 26–33% were not or very weakly expressed (<10 counts overall) and were discarded (Fig. 1A). In the end, between 34% (Zhang11) and 41% (Waters) of maize genes can be assessed for genomic imprinting per dataset and only 31% of all maize genes (12,354 genes) can be assessed in all datasets (Fig. 1A,B). In conclusion, even after consistent reanalysis, the set of genes that can be assessed for genomic imprinting differs considerably between the datasets. For all the following analyses, we considered only genes that could be evaluated for imprinting in all datasets.

**Figure 2:**
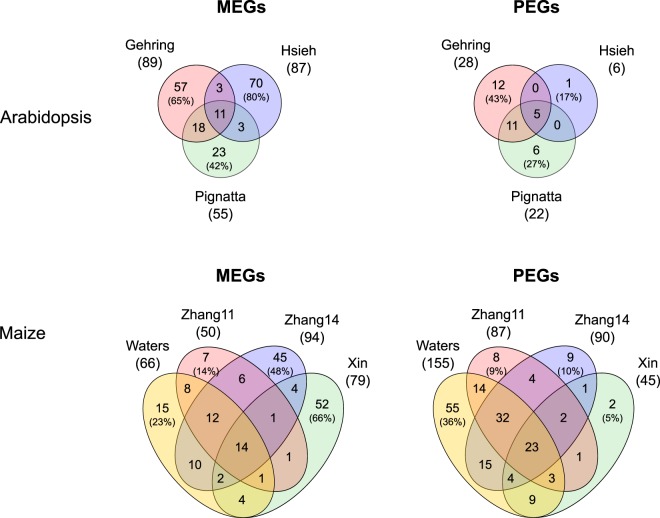
Venn diagrams showing the number of imprinted genes in hybrid endosperm reported by different studies in *Arabidopsis* L*er*/Col accessions and in maize B73/Mo17 inbreds. Only genes were considered that could be evaluated for imprinting in all datasets. Numbers in brackets denote the percentage of non-shared genes relative to the full set reported. The “Xin” and “Zhang11” sets comprise genes identified at 10 days after pollination and “Zhang14” comprises genes at 12 days after pollination. Genes with an accession/inbred-specific bias in expression were excluded from the analysis.

In *Arabidopsis*, a total of 185 genes that could be evaluated by all datasets were proposed to be maternally expressed imprinted genes (MEGs) in at least one study using the Landsberg *erecta* (L*er*) and Columbia-0 (Col-0) accessions (Fig. 2). The three studies proposed between 55 and 89 MEGs^28,29,38^. The large majority of genes (81%) were unique to a single study, and only eleven MEGs were identified as imprinted in all three studies (6%; Fig. 2). Thirtyfive genes were proposed to be paternally expressed imprinted genes (PEGs) in at least one study with five PEGs being commonly identified in all three studies (14%; Fig. 2). In maize, the four available studies using the inbreds B73 and Mo17^31,33,36,37^ listed 182 different MEGs and 182 PEGs (Fig. 2). The majority of genes (65% and 41% for MEGs and PEGs, respectively) was proposed by a single study only. The overlap between studies was also small, with 14 MEGs (8%) and 23 PEGs (13%) being commonly identified by all four studies.

The low concordance between studies could be due (i) to intrinsic, biological differences (e.g. developmental stage analyzed), (ii) to technical differences (particularly library preparation and complexity, sequencing depth and batch effects, reviewed in^41,42^, and/or (iii) to the varying bioinformatics/statistical analysis protocols applied. As summarized in Table 1, all studies differ in terms of developmental stage and some use different accessions creating considerable biological variation. Technical differences like library preparation, sequencing platform, read length, and single-end vs. paired-end sequencing introduce a further level of variation. Particularly, the observed differences in sequencing depth, expected read-mapping biases^48^, as well as in the completeness and quality of available SNP annotations, present likely technical sources of inconsistency (Table 1). Regarding statistical analysis, all studies applied count statistics (Table 1), which do not properly model count dispersion of RNA-Seq data, resulting in increased numbers of false positives particularly for large counts^41,44^. Furthermore, the studies apply very different criteria for filtering potentially imprinted genes according to the allelic bias (Table 1). The requirement to call a gene’s expression parentally biased differed tremendously between the studies and ranged from 90% of all reads that have to derive from one parent^31^, over 5 times more reads from one parent^33^, to simply assessing deviations from the expected 2:1 ratio in the endosperm^28^. Lastly, to deal with potential contamination from the seed coat, transcripts that were highly expressed in this maternal tissue were filtered out *in silico* in most studies (Table 1). The filtering conditions and the expression data used varied considerably across studies.

**Table 1:** Characteristics of data generation and data analysis of published genome-wide imprinting datasets in *Arabidopsis* and maize.

	Wolff 2011	Gehring 2011	Hsieh 2011	Pignatta 2014	Waters 2011	Zhang 2011	Xin 2013	Waters 2013	Zhang 2014
Organism	*Arabidopsis*	*Arabidopsis*	*Arabidopsis*	*Arabidopsis*	Maize	Maize	Maize	Maize	Maize
Strains	Col-0, Bur-0	Col-0, L*er*	Col-0, L*er*	Col-0, L*er*, Cvi	B73, Mo17	B73, Mo17	B73, Mo17	B73,Mo17,Ki11,Oh43	B73, Mo17
Starting Material	whole seeds	dissected endosperm/embryo	dissected endosperm/embryo	dissected endosperm/embryo	dissected endosperm/embryo	dissected endosperm/embryo	whole kernels (0,3,5 DAP)/dissected endosperm (7, 10,15 DAP)	dissected endosperm	dissected endosperm
Timepoint	4 DAP	6 – 7 DAP	7- 8 DAP	6 DAP	14 DAP	10 DAP	0,3,5,7,10,15 DAP	14 DAP	12 DAP
Biological Replicates per cross	1	1	2	3	1	1	1	1	1
Sequencing Platform	Illumina GAII	Illumina GAII	Illumina GAII	Illumina HiSeq	Illumina GAII/HiSeq	Illumina HiSeq	Illumina HiSeq	Illumina HiSeq	Illumina HiSeq
Read Length	36 bp	50/36 bp	76 bp	40/80 bp	2 × 76 bp	2 × 100 bp	2 × 90 bp	2 × 100/2 × 50 bp	2 × 100
NCBI SRA study ID	SRP005700	SRP007424	SRP003799	SRP033371	SRP009313	SRP011991	SRP026399	SRP031872	SRP011991
Genome annotation	TAIR 8	TAIR 9	TAIR 8	TAIR 10	B73 AGPv2	n/a	B73 5b.60	n/a	B73 (V2)
Total number of raw reads (hybrids)	122 mio	100 mio	165 mio	1,837 mio	245 mio	149 mio	379 mio	1,969 mio	154 mio
Total Number of SNPs	569,859	347,928	402,226	384,612	1.6 mio	51,416 exonic	6.5 mio	28,195–142,033 exonic	4.2 mio
**Mapping**									
Mapping software	vmatch	TopHat	Bowtie	Tophat v2.0.8	GSNAP	bwa	TopHat2	TopHat	TopHat
Number mismatches allowed	2/36	n/a	3/76	1/40	2/36	n/a	n/a	2	n/a
Allele-specific mapping bias	Alignment to Col and Bur pseudoreference (only SNPs)	n/a	Alignment to Col and L*er* pseudoreference (only SNPs)	n/a	n/a	Mapping to SNP-masked genes	Mapping to both genomes (reference-guided assembly of Mo17)	Mapping to SNP-masked transcripts (filtered gene set v5b.60)	Mapping to SNP-masked genes
**Counting and Statistics**									
Minimal coverage (allelic reads)	≥10 (≥30)	≥15	n/a	n/a	≥10 reads assigned to one allele in both hybrids	≥10 per cross	≥40 (non-stringent: ≥5 per cross)	≥10 per cross	n/a
Summing Reads across				gene	gene	individual SNPs	gene	gene	gene
Statistical Test	Binomial	Storer-Kim	Fisher’s exact test	Fisher’s exact test	Chi-Square	Chi-Square	Chi-Square	Chi-Square	Chi-Square
Multiple Testing Correction	yes (FDR 5%)	no (p < 0.01)	no (p < 0.001)	yes (FDR 1%)	no (p < 0.01)	no (p < 0.05)	unknown adjustment (p < 0.001)	no (p < 0.05 or p < 0.01)	no (p < 0.05)
Allelic bias filtering	n/a	only unique expression	MEGs: maternal score ≥ 4x paternal score; PEGs: paternal score ≥ 1.5x maternal score	MEGs: ≥ 85% maternal reads PEGs: ≥ 50% paternal reads	≥90% reads from one parent in both reciprocal crosses	≥83% reads from one parent	MEGs: ≥ 90% maternal reads PEGs: ≥ 70% paternal reads < 6 paternally derived reads at 0 DAP	Moderate MEGs: > 90% maternal reads PEGS: > 60% paternal reads. Strong MEGs/PEGs: > 90% maternal/paternal and p < 0.01	≥ 83% reads from one parent (reduced criteria: Chi-Square p < 0.05)
Filtering out potentially contaminating transcripts derived from the seed coat	Expression SLR in endosperm ≥ 3x seed coat and SLRs in vegetative tissues < 5 (less strigent criteria for low expressed genes)	MEGs expression in endosperm ≥ 2x higher than in the seed coat	Expression levels in endosperm < 4x than LCM-disected endosperm	Expression levels < 2x higher in the seed coat than embryo or endosperm	n/a	n/a	n/a	B73 expression atlas	n/a

Abbreviations: DAP, days after pollination; FDR, false discovery rate; LCM, Laser Capture Microdissection; MEG, maternally expressed imprinted gene; PEG, paternally expressed imprinted gene; SNP, single-nucleotide polymorphism; SLR, signal log ratio; SRA, short read archive (http://www.ncbi.nlm.nih.gov/sra).

### Analysis of Allelic Bias Using edgeR Outperforms other Methods

It was previously noticed that a large part of the differences between publications is owed to different statistical pipelines to call imprinted genes. When the two *Arabidopsis* datasets that analyzed the same accessions and a similar developmental stage and tissue (Gehring and Hsieh datasets) were analyzed in the same way, the overlap increased substantially (from 14 to 56 MEGs and from 6 to 18 PEGs^28^). Therefore, we created a statistical pipeline to identify genes with statistically significant allelic imbalance from the expected, endosperm-specific 2:1 ratio. Our pipeline is based on edgeR^45^ and analyzes counts by a generalized linear model (GLM), based on a negative binomial distribution in a paired design (parentals of the same cross) with two or more biological replicates (or reciprocal crosses). Importantly, edgeR models count overdispersion as shown exemplarily for the Pignatta dataset (Supplementary Fig. S1).

We then tested the performance of our statistical pipeline compared to other methods based on synthetic data, where we could control the settings and the true genomic imprinting status of each gene. We simulated counts using negative binomial distributions, with mean and dispersion parameters estimated from real data (see Material and Methods). Imprinted genes were introduced by adding 200 genes with a parent-of-origin-specific allelic bias: 50 MEGs each with strong (99% maternal reads) or moderate (85% maternal reads) allelic bias, as well as 50 PEGs each with strong (34% maternal reads) or moderate (48% maternal reads) allelic bias. Random allelic read sampling was modeled by sampling from a binomial distribution.

We evaluated the performance of three different methods: our edgeR-based pipeline, Fisher’s exact test, and Stouffer’s method. Fisher’s exact test is the analysis method used in most published plant studies where a Fisher’s exact test is performed with allelic counts of summed reciprocal crosses. From now on this method will be called “Fisher-summed”. Stouffer’s method^49^ is a method to combine p-values bearing the same null hypothesis. We combined the two p-values per gene calculated by Fisher’s exact tests comparing to expectations for each reciprocal cross separately, and calculated a combined adjusted p-value using Stouffer’s method. From now on this method will be called “Fisher-combined”.

**Figure 3:**
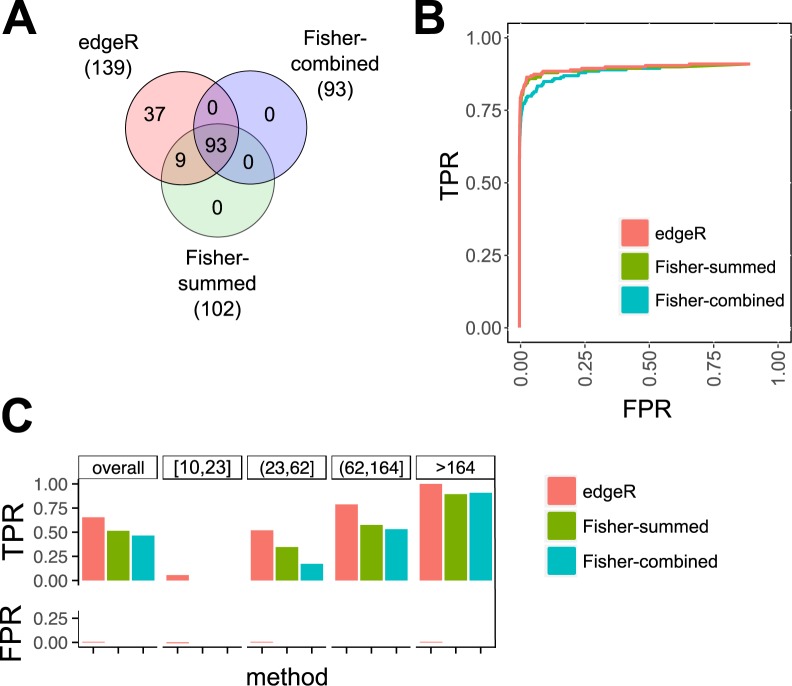
Benchmarking of three tested methods to identify imprinted genes using simulated data. (**A**) Overlap of detected spike-in imprinted genes between different methods (FDR 5%). (**B**) ROC curves. (**C**) True positive rates (TPR) and false positive rates (FPR) across four equally sized categories of genes with increasing expression levels (number of counts) at FDR 5%.

In the simulation setting, our pipeline (edgeR) identified the largest number of observed true positives (spiked-in MEGs and PEGs) (Fig. 3A), identifying 131 true positives (of 200, 66% true positive rate [TPR]) with 8 false positives (falsely detected as imprinted), whereas the second-best method, Fisher-summed, identified only 102 true positives (51% TPR) with 0 false positives (0% FPR). Fisher-combined was close third, identifying 93 true positives (47% TPR) with 0 false positives (0% FPR).

The Venn diagram (Fig. 3A) shows that 93 genes were identified between all three methods, 9 were shared between edgeR and Fisher-summed, 37 genes were only identified by edgeR, and 61 true positive genes were not identified by any method. Considering the genes that were simulated to be imprinted as the true positive group, and the remaining genes as the true negative group, we computed the false positive rate and the true positive rate for all possible score thresholds and constructed a ROC (Receiver Operating Characteristic) curve for each method (Fig. 3B). edgeR and Fisher-summed had a small performance advantage in detecting the spike-in genes over Fisher-combined. We divided the assessed genes into four equally sized bins according to their number of counts. As expected, the true positive rate (TPR) increased with larger counts per gene for all methods (Fig. 3C; Supplementary Fig. S2). TPR was highest for edgeR in all categories with a large advantage in the two categories with the lowest counts, while the other two methods only passed the 50% TPR for the two quartiles of genes with the largest counts. True positive rates also depended on the degree of allelic imbalance: at a 5% False Discovery Rate (FDR) cut-off, edgeR achieved TPRs of 80% and 54%, respectively, for strong and weak MEGs, and TPRs of 70% and 58%, respectively, for strong and weak PEGs. In contrast, the second best method Fisher-summed achieved TPRs of 74% and 44%, respectively, for strong and weak MEGs, and TPRs of 58% and 28%, respectively, for strong and weak PEGs. Simulations with count distributions similar to the largest observed datasets showed that edgeR still performed better than the other methods (data not shown). EdgeR outperformed the other methods when 200 genes with an accession-specific bias in expression were added to the 200 simulated imprinted genes (data not shown). In addition, the performance of edgeR was robust against varying proportions of simulated strongly and moderately expressed imprinted genes (Supplementary Table S4) and against varying numbers of imprinted genes, as long as the total number did not drop below 50 genes (Supplementary Table S5).

### Consistent Reanalysis of Published Imprinting Studies with edgeR Identifies More Common Imprinted Candidate Genes in *Arabidopsis* but not in Maize

**Figure 4:**
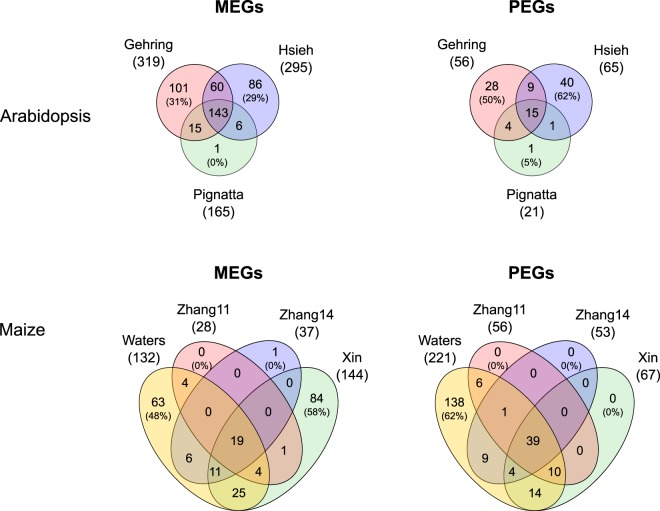
Venn diagrams showing the overlap between imprinted candidate genes across datasets when reanalyzing the raw data using the same standardized method with generalized linear models/edgeR at a FDR cut-off of 5%. Only genes were considered that could be evaluated for imprinting in all datasets. Numbers in brackets denote the percentage of non-shared genes relative to the full set detected in the dataset. The “Xin” and “Zhang11” sets comprise genes identified at 10 days after pollination and “Zhang14” comprises genes at 12 days after pollination.

To test our statistical pipeline on real data, we identified genes with statistically significant allelic imbalance from the expected, endosperm-specific 2:1 ratio using edgeR^45^ with reanalyzed data starting from raw reads. We identified between 165–319 candidate MEGs and 21–65 candidate PEGs for the examined datasets using a 5% FDR cut-off (Fig. 4). Diagnostic plots exemplary for the Pignatta dataset display a good model fit (Supplementary Fig. S3). A list of the called imprinted candidate genes in *Arabidopsis* and maize can be found in Supplementary Tables S1 and S2, respectively.

In *Arabidopsis*, we identified a larger number of potentially imprinted genes with clearly increased overlaps: 143 common MEGs were found from all three datasets (87% of the smallest dataset [Pignatta], and 45% of the largest dataset [Gehring]) and, in addition, 60 MEGs (20% of the smaller dataset) were shared by the Gehring and Hsieh datasets (Fig. 4). Now only 31% and 29% of identified MEGs were unique to the Gehring and Hsieh datasets, respectively. In the Pignatta dataset, only a single MEG (1/165, 0%) and a single PEG (1/21, 5%) were not shared with any other dataset, potentially owing to the fact that (i) the Pignatta dataset had the largest sequencing depth and therefore the largest gene coverage, and (ii) three biological replicates per cross could be analyzed, allowing the identification of high-confidence candidates with a small number of false positives. We also identified an almost three-fold increased number of PEGs (98 instead of 35 genes as originally published), although with an increased proportion of non-shared PEGs in the Hsieh dataset (from 17% [1/6] to 62% [40/65]).

In maize, we identified a large number of imprinted candidate genes, 218 MEGs and 221 PEGs (Fig. 4). 70 MEGs (32%) and 83 PEGs (38%) were identified from at least two different datasets, leading to an overlap of MEGs similar to the one found in the originally published lists (Fig. 2). The number of candidate imprinted genes varied a lot between datasets, limiting the maximum number of genes shared by all four datasets. The proportion of non-shared MEGs and PEGs decreased for all datasets, except for Waters MEGs and PEGs, which increased from 22% to 48% and from 36% to 62%, respectively, likely due to the significant increase in the number of imprinted candidate genes (Fig. 4). In contrast, the Zhang datasets, as well as the PEGs identified from the Xin dataset, showed almost no exclusive candidate imprinted genes.

Furthermore, we identified the top50 imprinted candidate genes, which are statistically most significant for each dataset and compared the number of shared genes (Supplementary Fig. S4). Between 17–28 candidate genes were common to all compared datasets and the proportion of non-shared candidates decreased or was similar as in the originally published lists except for the Hsieh, Pignatta, and Xin PEGs.

In summary, a reanalysis of the datasets using our edgeR-based pipeline produced gene lists with a clearly larger overlap in *Arabidopsis*, despite identifying a larger number of imprinted candidate genes with less pronounced allelic imbalance. Thus, the Jaccard similarity indices between *Arabidopsis* datasets were higher for MEGs and identical for PEGs after reanalysis with edgeR compared to the originally published gene lists (Supplementary Table S6). For maize, we saw an increase in dataset concordance after reanalysis for both MEGs and PEGs.

### Reanalysis Using the edgeR Analysis Pipeline Identifies Novel Imprinted Candidate Genes in Comparison to the Original Analysis

**Figure 5:**
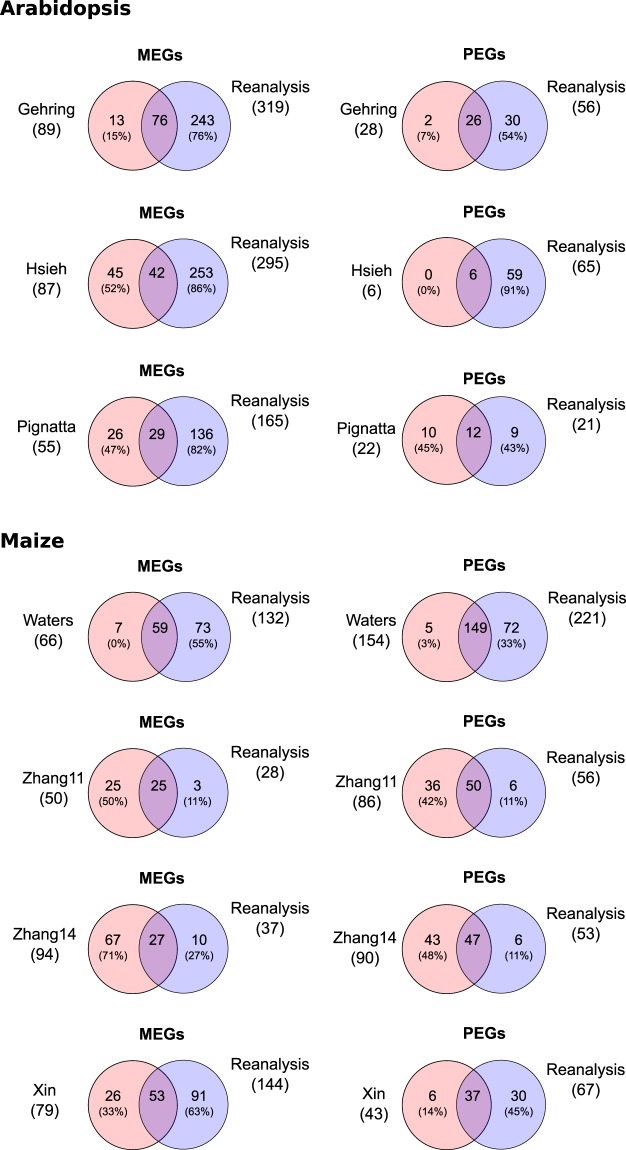
Pairwise comparison of imprinted genes between the originally published analysis and the reanalysis using generalized linear models and edgeR with a 5% FDR cut-off. Only genes were considered that could be evaluated for imprinting in all datasets. Numbers in brackets denote the percentage of non-shared genes relative to the full set detected in the dataset. For the “Xin” dataset only one timepoint (10 days after pollination) is shown. The “Zhang11” set comprises genes identified at 10 days after pollination and “Zhang14” comprises genes expressed at 12 days after pollination.

Having identified imprinted candidate genes using our new analysis pipeline, we compared the candidate genes pairwise with the lists from the original publications (Fig. 5). When comparing with our candidate MEGs and PEGs at a 5% FDR cut-off, 0–71% of previously published imprinted candidate genes were also identified by our pipeline.

From the reanalysis we selected the genes with topmost significance to get the equivalent number of the previously published imprinted candidate genes and compared them to the original publications. *Arabidopsis* PEGs and maize MEGs and PEGs generally overlapped at 44–85% between the reanalysis and the original gene lists (Supplementary Fig. S5), much higher than for *Arabidopsis* MEGs, where often only negligible overlaps were observed.

In conclusion, the reanalysis with a standardized analytical pipeline identifies a large number of similar candidates but also additional novel candidate genes. Furthermore, it fails to identify previously called imprinted candidates, likely owing to the improved statistical analysis, which takes count overdispersion into account and neglects large allelic biases in transcripts covered by a low number of reads.

### Consistent Reanalysis Using Fisher’s Exact Test Identifies Fewer Overlapping Imprinted Candidate Genes

We also reanalyzed the data from the seven studies using the “Fisher-summed” method in order to assess its performance with real data. We selected the genes with topmost significance to get for each dataset the equivalent number of the previously published imprinted candidate genes (Supplementary Fig. S6). A proportion of maternal reads of ≥85% and ≤50% was required for candidate MEGs and PEGs, respectively. In *Arabidopsis*, the number of genes identified from all three datasets (27 MEGs and 1 PEGs; Supplementary Fig. S6) was only a fraction of those identified after reanalysis with edgeR (41 MEGs and 5 PEGs; Supplementary Fig. S7). Also, the number of genes shared between at least two different datasets (63 MEGs and 10 PEGs; Supplementary Fig. S6) was smaller than after reanalysis with edgeR (70 MEGs and 19 PEGs; Supplementary Fig. S7). The proportion of non-shared genes was 29–68% per dataset, much higher than after reanalysis with edgeR, where proportions of non-shared genes were 4–32%. A reanalysis of the maize datasets using the “Fisher-summed” method produced a similar concordance between datasets as edgeR (Supplementary Figs S6 and S7). Accordingly, Jaccard similarity indices between datasets were markedly higher in *Arabidopsis* after reanalysis with edgeR for MEGs compared with the Fisher-summed reanalysis or the originally published gene lists (Supplementary Table S6). For PEGs, Jaccard similarity indices after reanalysis with edgeR were higher than after Fisher-summed reanalysis and identical with the originally published gene lists.

### Power of Detecting Imprinted Genes Is Relatively Small due to Non-saturating Sequencing Depth

**Figure 6:**
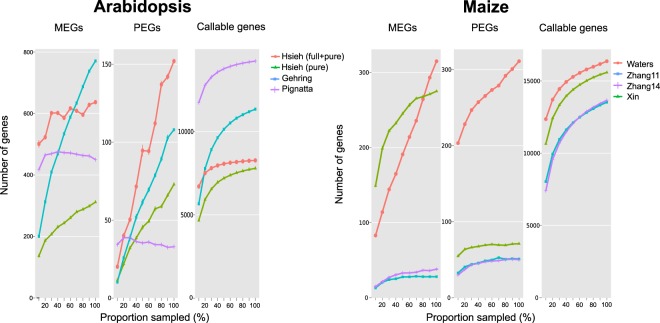
Saturation curves showing numbers of detected MEGs and PEGs from various datasets. The curves were generated by randomly sampling increasing proportions of each dataset and identifying imprinted candidate genes using the same pipeline. Values are means (and standard errors) of 10 random subsamples.

The power-sample size relationship in allelic imbalance detection is not well understood as it depends mainly on the degree of allelic bias, the sequencing read length and coverage (expression strength), but also on the detection procedure. We show that sequencing depth is far from being saturated, as shown by random subsampling of each dataset and detecting MEGs and PEGs by our edgeR-based pipeline (Fig. 6). For most datasets, the number of detected MEGs and PEGs do flatten to some extent with increasing sampling proportions. The low numbers of detectable genes in the Zhang 11 and Zhang 14 are likely due to low mapping rates and low sequencing depth. The relatively flat slopes for the total number of callable genes indicate that a huge increase in sequencing depth would be required to assess the remaining 4–10% genes that were expressed, but did not reach the minimal read coverage of 10 reads.

## Discussion

### Reanalysis of Imprinting Studies Reveals that Biological and Technical Differences Strongly Contribute to Biases in Identifying Imprinted Genes

Published studies strongly disagree in the extent and composition of imprinted genes in the endosperm (Fig. 2). We reanalyzed both simulated and real data from seven genome-wide imprinting studies in *Arabidopsis* and maize, using a standardized bioinformatics pipeline based on generalized linear models. In *Arabidopsis*, our analysis identified an increased number of imprinted genes co-identified by at least two different datasets, particularly for MEGs where the overlap between datasets was strikingly larger (Fig. 4 and Supplementary Fig. S4). In maize, consistent reanalysis identified a slightly increased number of imprinted genes shared between studies both for MEGs and PEGs. For MEGs, it is conceivable that the increased concordance is partially due to the fact that we did not filter out potential seed coat contamination in the reanalysis. However, as it has previously been shown that most imprinted genes in the embryo are also expressed in the seed coat^50^, such a filter could also eliminate truly imprinted genes.

It was previously proposed that the discrepancies might at least in part stem from different data analysis pipelines to call imprinted genes and, in fact, using similar filtering conditions produced more overlap^28,34^. However, even after consistent reanalysis, a high number of imprinted candidate genes are unique to a single dataset, and reanalysis is able to increase the overlap across datasets only by a limited degree. This suggests that biological variation (such as different environmental growth condition, different developmental seed stage, stochastic allelic expression differences), technical differences (e.g., library preparation/complexity, batch effects^41,42^), and differences in sequencing depth inherent to the datasets contribute to these discrepancies and cannot be corrected for *in silico*.

Particularly in maize, reanalysis did not increase the concordance between datasets. Even though the developmental seed stages did vary to some extent (Table 1; 10–14 DAP), we cannot fully explain this finding by biological variation only. All original analyses used the same statistical test and highly similar conditions for allelic bias filtering. Possibly, the published original analyses of maize datasets used filtering conditions close to the optimum, such that edgeR-based reanalysis could not further improve concordance. Furthermore, the co-identification of imprinted genes is additionally hampered by unequal read coverage: 48 of 129 imprinted genes proposed by Zhang and colleagues^33^ had too few reads to characterize imprinted expression in the data of Waters and colleagues^31^. The relative importance of biological *versus* technical biases on the large discrepancy between the datasets from maize is currently difficult to assess.

### A Statistical Approach that Uses Generalized Linear Models and Takes Replicate Information into Account Largely Increases Sensitivity

Our statistical approach involves modeling of count data and is quite different from the approach chosen by the authors in the original publications. Our approach relies on generalized linear models and edgeR, which is a commonly used, well-established and robust method for differential expression analysis of RNA-seq data. By interpreting allelic counts of a cross as separate samples and imprinting analysis as a differential gene expression problem, we benefit from the power and flexibility of generalized linear models based on edgeR. The method is highly flexible, i.e. also batch effects can be modeled. The general methodology used here is also applicable to imprinting analysis in other tissues, where a deviation from 1:1 is tested. A further advantage of our statistical approach is that it circumvents somewhat arbitrary minimum cut-offs of effect size (allelic imbalance), which would require an individual optimization for each dataset. Most genes are only partially imprinted and we believe that the current knowledge does not support the exclusion of genes with moderate allelic imbalance yet reaching statistical significance. Our approach also allows (nearly) genome-wide ranking of genes according to their likelihood of being imprinted, allowing downstream applications, such as gene set enrichment analysis, with increased statistical power (e.g. for Gene Ontology analysis).

Prior to read counting, many steps are required to map, filter, and count allelic reads. To assess the importance of the preprocessing/counting relative to statistics, we reanalyzed the original allelic counts from^28^ using our method. We found a large discrepancy for MEGs and PEGs with original gene lists, whereas MEGs and PEGs largely agreed with the gene lists obtained from complete reanalysis with our analysis pipeline (data not shown). Given the large discrepancy, the statistical approach seems to have a larger relative contribution than the earlier steps required to obtain the variant counts.

### Simulation Reveals that edgR-based Analysis Outperforms Other Methods

The edgeR-based approach performed well in our simulation setting and clearly outperformed the other methods we tested. Notably, the other methods based on Fisher’s exact test seem overly conservative and missed most spike-in imprinted genes. edgeR was the only method to predict false positives due to a relatively poor FDR control for the lowest quartile of counts (10–23 counts per gene) where 4 of edgeR’s totally 8 false positive imprinted genes were identified. edgeR’s specificity could be further increased by selecting a larger minimal number of counts per gene (e.g., a cut-off of 20 allelic counts per gene). In our simulation setting, edgeR’s sensitivity was highest at a minimal cut-off of 10 or 20 reads with TPRs of 0.66 and 0.65, respectively, but dropped markedly at a cut-off of 50 reads with a TPR of 0.54 (Supplementary Table S7).

Importantly, our simulation did not aim to model biological variability predicting biological replicates. All biological systems have inherent biological variation and edgeR can account for it, whereas Fisher’s exact test completely ignores the within-condition variability, as it requires counts from replicates to be summed up for each condition. Therefore, we expect that edgeR would outperform methods using Fisher’s exact tests even more strikingly when biological replicates are available.

### Higher Coverage, Replicate Samples, and edgeR-based Analysis Could Improve the Identification of Imprinted Genes

The first generation of genome-wide studies identified many new imprinted genes in plants, yet a considerable proportion of genes could not be characterized due to low sequencing depth or insufficient genetic heterogeneity between the parents. Future experiments in genomic imprinting will be performed using paired-end reads with high sequencing coverage. With the rapid development in NGS, higher coverage is now readily achievable, which will notably increase the statistical power to detect imprinted genes. In the studies reanalyzed here, low counts were observed for many genes close to the minimal coverage cut-off of 10, where the variance is large and a large allelic bias is required to reach significance. Importantly, a sufficient number of biological replicates (i.e., at least three per reciprocal cross^51^) would permit to reliably estimate the variability from the data, enabling the performance of a more robust differential expression analysis and a more reliable estimation of the total number of imprinted genes.

### Biological Perspectives

When comparing datasets from different studies, we assume that the same set of genes is imprinted over the sampling time period. Partial or complete violations of the assumption also decrease the amount of overlap across datasets in addition to technical and analytic biases. Indeed, first studies showed dynamic expression of imprinted genes in maize^36^. Furthermore, we cannot solely rely on statistical significance in calling a gene imprinted or not. There are several ways to prioritize a gene list after statistically calling genes with an allelic bias in a given tissue. First, filtering the gene lists by fold difference between the two parental alleles could help to identify genes that can more easily be validated experimentally. Second, filtering the gene lists against tissue-specific expression data from seeds might identify genes relevant to the tissue of interest^52,53^. However, expression of a given gene in other (e.g. maternal seed coat) tissues does not exclude an allelic bias in the fertilization products^50^. Third, comparing the gene list against central cell and sperm cell expression data^54–56^ can inform to what extent the allelic bias is a result of expression in the fertilization products or might at least partially represent carry-over of gametic transcripts that were produced prior to fertilization.

An improved assessment including larger gene sets (by using different sets of strains), as well as imprinted genes with moderate allelic imbalance, will provide further insights into the extent and biological significance of genomic imprinting. Having the full catalog of imprinted genes in several plant species will also allow the tackling of evolutionary questions about genomic imprinting, including its origin and fixation, the conservation of imprinted genes, and their gain and loss of imprinting status on the phylogenetic tree.

Lastly, we have to stress that without further confirmation, gene lists are not representative with regard to the absolute number of imprinted candidate genes expressed in the endosperm. Considering this, it is indispensable to confirm bioinformatically identified imprinted candidate genes by alternative methods, such as allele-specific expression analysis using RT-PCR and Sanger sequencing, pyrosequencing, and/or reporter gene assays.

## Conclusions

A new, effective data analysis pipeline is reported that allows for an improved analysis of RNA-seq data from reciprocal F1 crosses. The pipeline allows for the modelling of biological replicates and is applicable to any diploid or triploid tissue. Furthermore, our genome-wide ranking of *Arabidopsis* and maize imprinted candidate genes, which integrates all available datasets, provides a useful resource to inform future experiments focused on understanding genomic imprinting in plants.

## Methods

### Read Mapping and Counting

FASTQ-formatted raw reads were downloaded from the NCBI Short Read Archive (SRA) for endosperm experiments for *Arabidopsis* (GSM674847, GSM674848, GSM756822, GSM756824, GSM607727, GSM607728, GSM607732, GSM607735, GSM1276498, GSM1276500, GSM1276502, GSM1276504, GSM1276505, GSM1276508, GSM1276509, GSM1276512, GSM1276514, GSM1276515) and for maize (SRX105679, SRX105678, SRX114629, SRX114630, SRX047539, SRX047544, SRP031872, GSE48425). Hsieh samples obtained through laser capture microdissection were not included in the analysis as their inclusion reduced the overlap with other datasets (data not shown). Reads were quality-checked with the FastQC application (http://www.bioinformatics.babraham.ac.uk/projects/fastqc/). To reduce the bias in mapping reads towards reference alleles, we aligned the reads to a masked reference genome, in which bases at known polymorphic sites were replaced with “N”. Reads were mapped to the genome using STAR v2.3.0e^57^. Only reads that mapped to a unique position in the genome were considered for further analysis.

For *Arabidopsis* the genome annotation TAIR10 (http://www.arabidopsis.org/) was used, and for maize AGPv3 annotated genes were downloaded from Ensembl Plants Release 31 (http://plants.ensembl.org/)^58^. *Arabidopsis* SNP annotation files were obtained from the 1001 Genomes project^59^ http://1001genomes.org/data/MPI/MPISchneeberger2011/releases/2012_03_14/). Maize hapmap v3.2.1 SNP variants^60^ in VCF format (Release date 3/3/2016) were obtained from the Panzea database (http://cbsusrv04.tc.cornell.edu/users/panzea/download.aspx?filegroupid=15) and filtered for high-confidence (flag LLD) biallelic SNP variants, which were polymorphic between B73 and Mo17 and homozygous in both inbred strains. Allelic reads were counted at previously identified SNP positions between homozygous parentals, using Python v3.2 with pysam v0.8.4^61^, while positions with low sequencing quality (phred quality <20) were excluded. No more than one SNP was counted per read to prevent pseudo-replication. Counts were summed up per gene and variants with less than 10 reads (summed across the two reciprocal crosses) were discarded.

### Testing for Allele-specific Expression

To assess allele-specific expression, we used edgeR version 3.4.2^45^. It uses an empirical Bayes estimation based on the negative binomial distribution. For library size normalization and to eliminate composition biases between libraries, we used the TMM (Trimmed Mean of M-values) method. TMM normalization keeps the ratio between maternal and paternal allelic reads in a cross at approx. 2 (data not shown). Experiments were analyzed using a generalized linear model with a paired design (the two allelic counts of the same cross treated as paired samples) and at least two biological replicates (the two reciprocal crosses). When biological replicates were available, they were included in the model. We used tagwise dispersion estimates. False Discovery Rate (FDR) was calculated according to Benjamini and Hochberg^62^. The R software version 3.0.3^63^ was used for statistical analysis and for creating the graphs.

### Simulation of genomic imprinting

To generate synthetic data we used the function makeExampleDESeqDataSet of the DESeq2 version 1.2.10^46^ R/Bioconductor package. Mean and dispersion parameters that were used in the simulation were estimated from real RNA-seq data (interceptMean = 2, interceptSD = 3). Two biological replicates were simulated to serve as the two reciprocal crosses. No outlier counts and differential expression were introduced. The total number of genes in each simulated dataset was 15,000, and their true proportion of maternal reads was set to 2/3. Then we randomly picked 200 genes and modified their imprinting status, 50 each of strong MEGs (99% maternal reads), weak MEGs (85% maternal reads), strong PEGs (34% maternal reads) and weak PEGs (48% maternal reads). Random allelic read sampling was modeled by sampling from a binomial distribution with the probability of success set to the true proportion of maternal reads.

We evaluated three methods for identifying genes with parent-of-origin-specific expression: edgeR, Fisher’s exact test, and Stouffer’s method. Fisher’s exact test does two separate statistical tests for the two reciprocal crosses, and the resulting two p-values per gene were combined with Stouffer’s method (calculated by sumz function of R package metap). Benchmarking was performed using Venn diagrams and Receiver Operating Characteristic (ROC) curves with iCOBRA^64^ https://github.com/markrobinsonuzh/iCOBRA.

### Comparison with Published Gene Lists

Published gene lists were compiled from the Supplementary Data of the respective publications. For the Waters B73xMo17 comparison, the updated list from^35^ was used, not the original one^31^. If lists of imprinted genes were available at various stringencies, we used the least stringent list (e.g. moderately imprinted genes for the Waters dataset). Genes with an accession/inbred-specific bias in expression were omitted. The Zhang14 dataset comprised only the lists of 106 MEGs and 91 PEGs at 12DAP (Prof. Xiaomei Lai, personal communication) described in^37^ but not the endosperm samples at 10DAP which were already described in^33^ and contained in the Zhang11 dataset. Genes that could not be evaluated for imprinting in all datasets were filtered out.

### Saturation Plots

In order to assess the degree of undersampling, we performed random subsampling on the count data and performed the same processing and statistical analysis steps as for the full data.

## Electronic supplementary material

Supplementary Figures S1-S7

Supplementary Table S1

Supplementary Table S2

Supplementary Tables S3-S7

## Data Availability

Scripts and data used in this manuscript are available on github (http://www.github.com/swyder/Reanalysis_plant_imprinting) and as supplementary files.
