# Supplementary material for: Consistent Reanalysis of Genome-wide Imprinting Studies in Plants Using Generalized Linear Models Increases Concordance across Datasets
Source: Sci Rep. 2019 Feb 4;9:1320. doi: 10.1038/s41598-018-36768-4 (PMC6362150; doi:10.1038/s41598-018-36768-4)
Supplement: Supplementary file 1 — Supplementary Figures S1-S7 [file 41598_2018_36768_MOESM1_ESM.pdf]

Consistent Reanalysis of Genome-wide Imprinting Studies in Plants Using Generalized Linear Models Increases Concordance across Datasets

Stefan Wyder, Michael T. Raissig, Ueli Grossniklaus

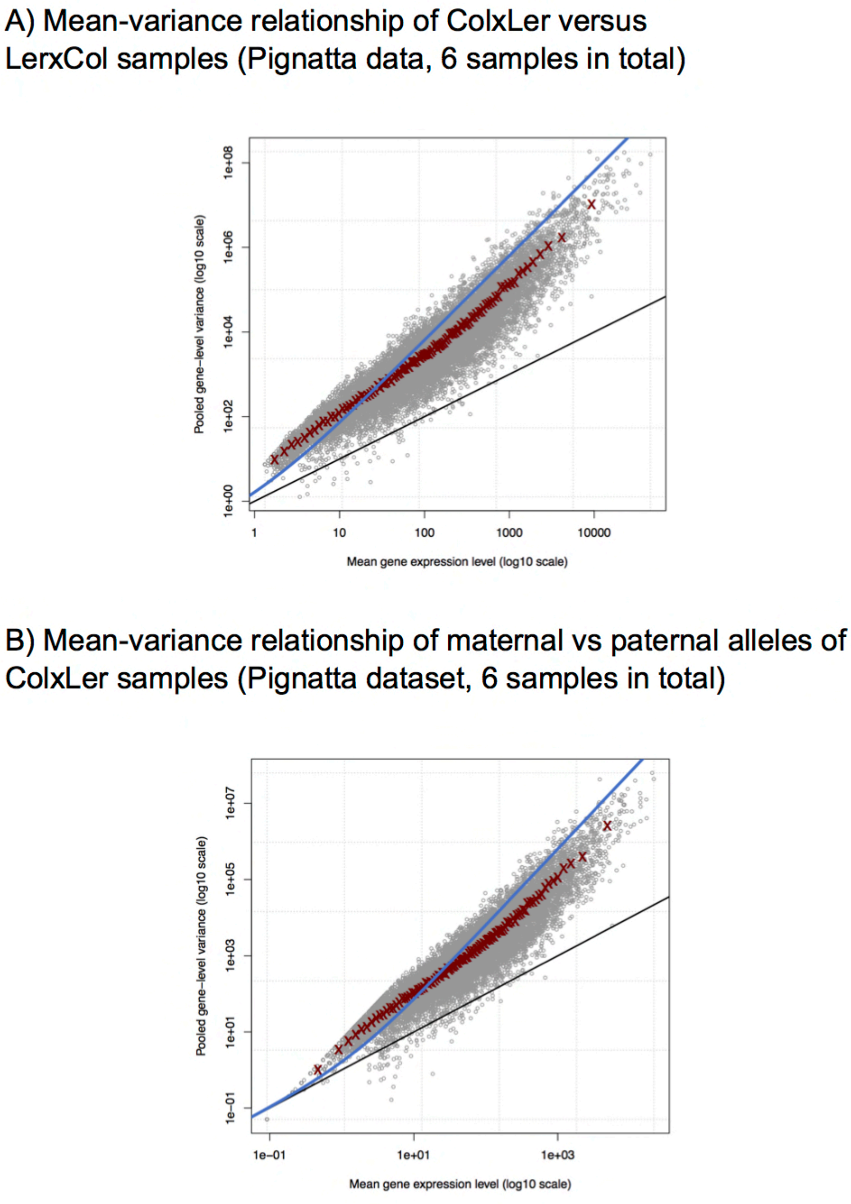

Supplementary Figure S1. Count overdispersion in the Pignatta dataset. Mean and variance are plotted for A) ColxL*er* versus L*er*xCol samples (3 samples each) and B) maternal versus paternal samples in ColxL*er* crosses (3 samples each). The blue line shows the Negative Binomial model with common dispersion and the black line shows the Poisson mean-variance relationship. The best fit with the data is observed by averaging raw variance for tags split into bins by overall expression level (red crosses).

In both plots the variance for the counts between samples is much larger than the mean, indicating overdispersion.

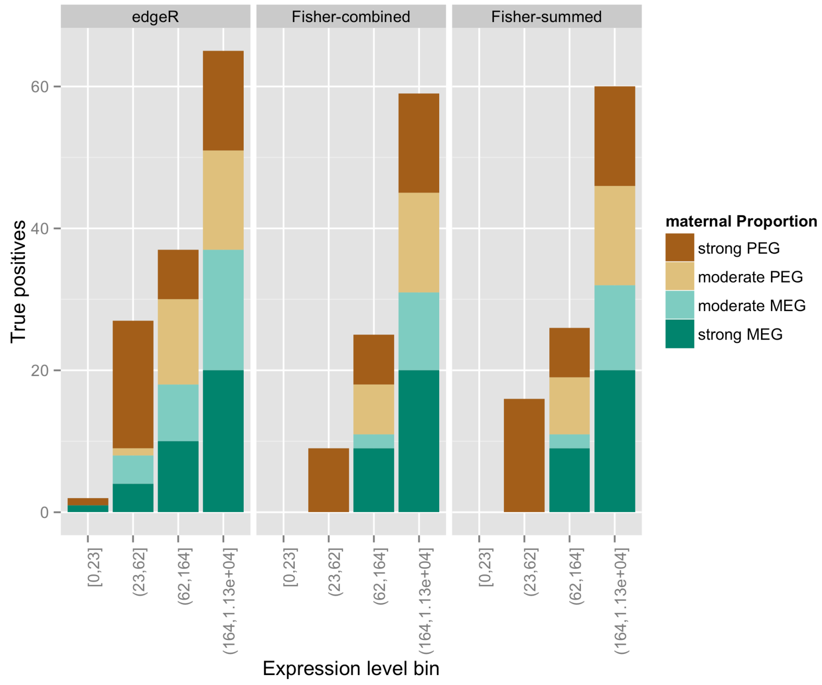

Supplementary Figure S2. Benchmarking of three tested methods to identify imprinted genes using simulated data. True positive genes (TPR) across four equally sized categories of genes with increasing expression levels (number of counts) at FDR 5%.

A) Plot of genewise biological coefficient of variation (BCV) against gene abundance (in log2 counts per million). The common, trended and tagwise BCV estimates are shown

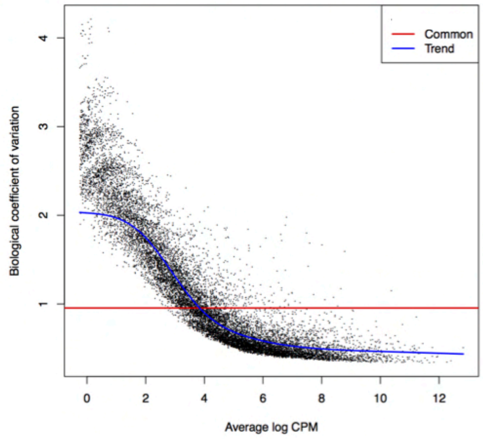

B) MA Plot showing the log-fold change (y-axis) against the average gene abundance (in log2 counts per million)

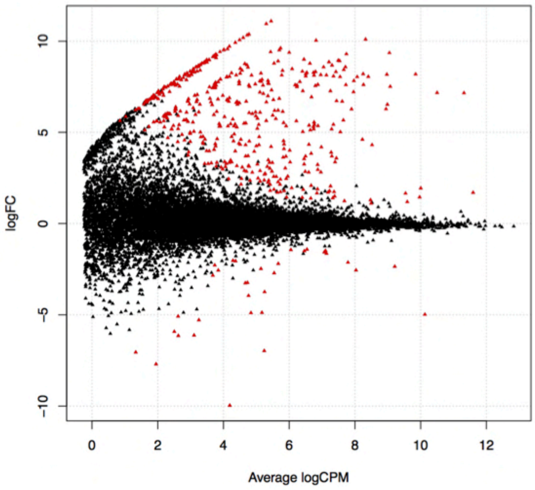

Supplementary Figure S3. Diagnostic plots for the Pignatta dataset. A) Genewise biological coefficient of variation (BCV) against gene abundance (in log2 counts per million). B) MA plot of the Pignatta dataset. Genes with a significant allelic bias at a 5% FDR cut-off are highlighted in red.

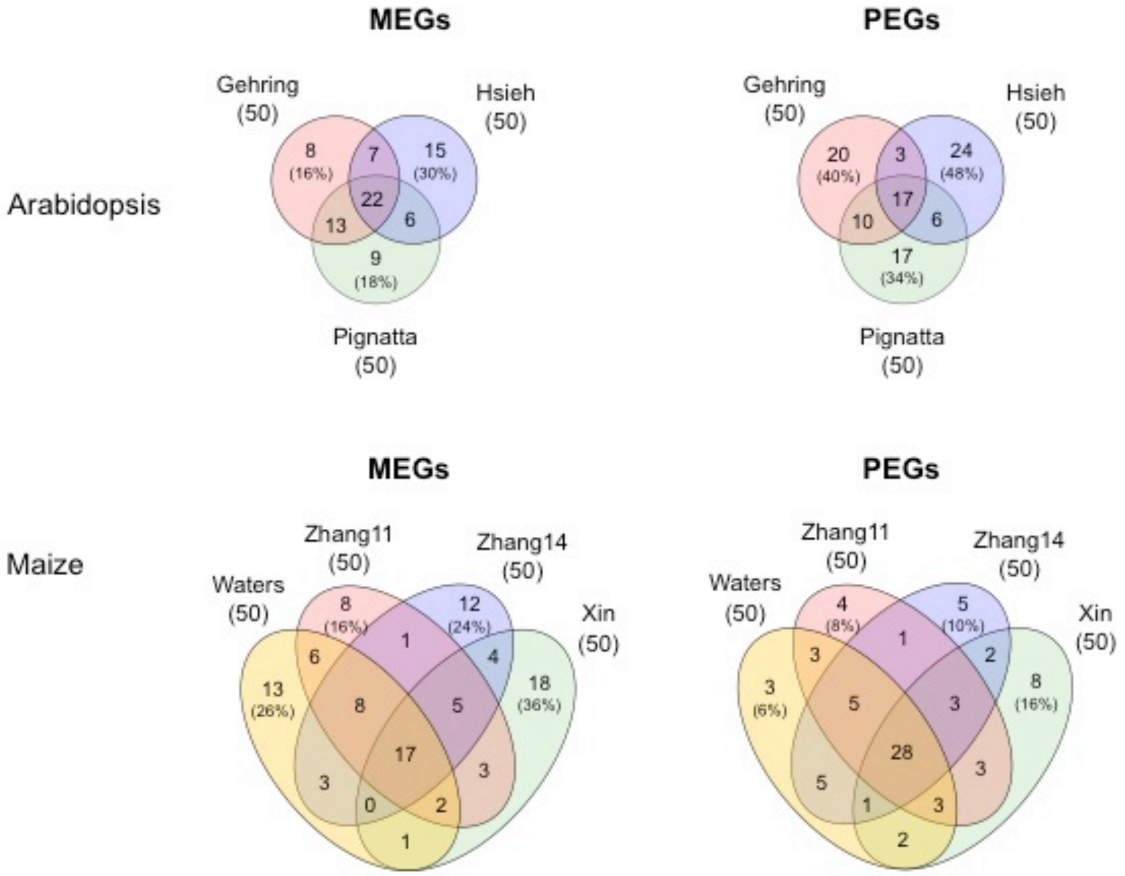

Supplementary Figure S4. Venn diagrams showing the overlap between the top50 imprinted candidate genes across datasets when reanalyzing the raw data using the same standardized method, using generalized linear models and edgeR. Only genes were considered that could be evaluated for imprinting by all datasets. Numbers in brackets denote the percentage of non-shared genes relative to the full set detected in the dataset.

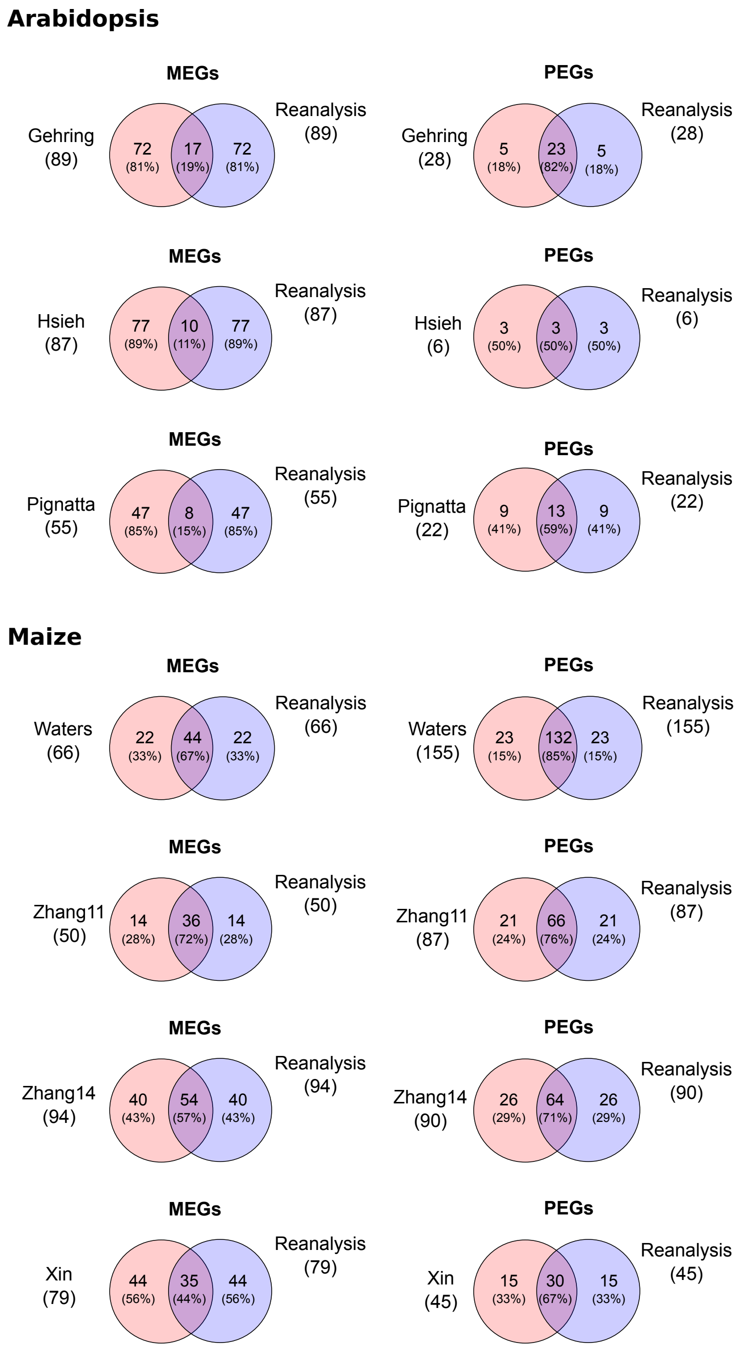

Supplementary Figure S5. Pairwise comparison of imprinted genes between the originally published analysis and the reanalysis using generalized linear models and edgeR. The same numbers of topmost imprinted genes were selected from the datasets reanalyzed with generalized linear models and edgeR. Only genes were considered that could be evaluated for imprinting by all datasets. Numbers in brackets denote the percentage relative to the full set.

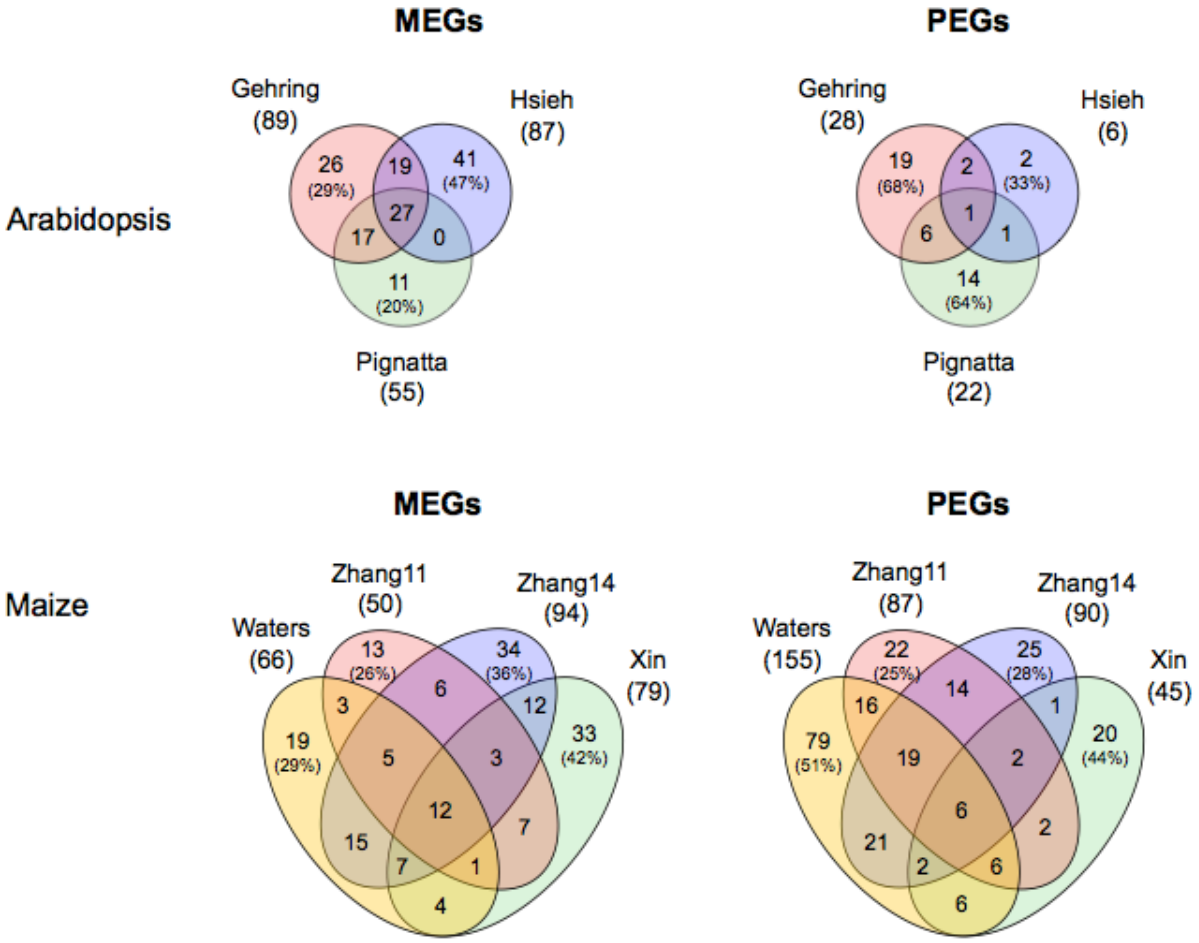

Supplementary Figure S6. Venn diagrams showing the overlap between imprinted candidate genes across datasets when reanalyzing the raw data using the "Fisher-summed" method with a maternal read proportion of at least 85% for MEGs and of at most 50% for PEGs. For each dataset the same numbers of topmost imprinted genes were selected from the reanalyzed datasets as previously published. Only genes were considered that could be evaluated for imprinting by all datasets. Numbers in brackets denote the percentage of non-shared genes relative to the full set detected in the dataset.

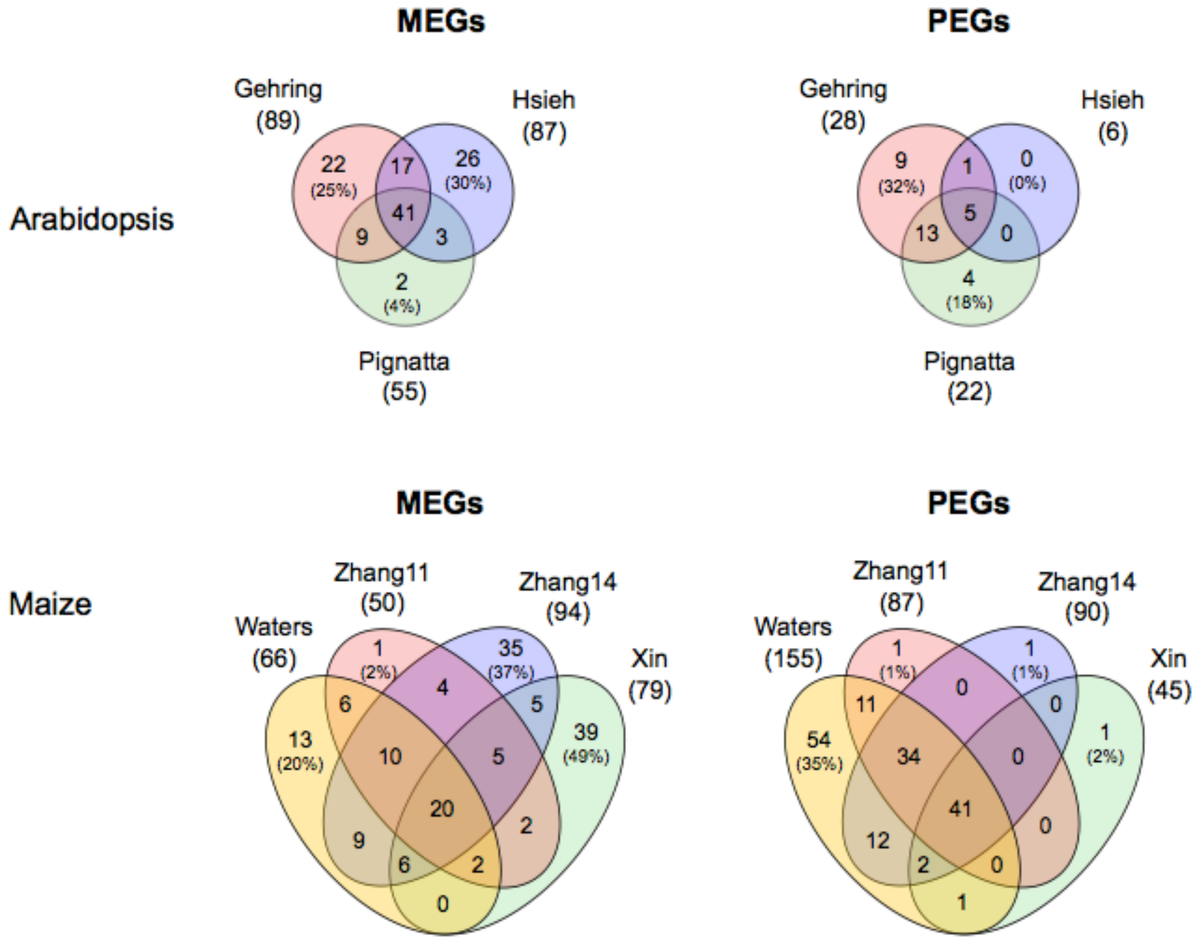

Supplementary Figure S7. Venn diagrams showing the overlap between imprinted candidate genes across datasets when reanalyzing the raw data using generalized linear models and edgeR. For each dataset the same numbers of topmost imprinted genes were selected from the reanalyzed datasets as previously published. Only genes were considered that could be evaluated for imprinting by all datasets. Numbers in brackets denote the percentage of non-shared genes relative to the full set detected in the dataset.

Supplementary Table S1. List of candidate imprinted genes identified in this study for *Arabidopsis*. Genes are sorted with decreasing probability of being imprinted in Gehring, Hsieh, and Pignatta datasets.

Supplementary Table S2. List of candidate imprinted genes identified in this study for maize.

Supplementary Table S3. Effect of allelic imbalance on edgeR sensitivity and specificity at 5% FDR in simulated data.

Supplementary Table S4. Effect of varying proportions of simulated strongly and moderately imprinted genes on edgeR sensitivity and specificity at 5% FDR.

Supplementary Table S5. Effect of varying numbers of simulated imprinted genes (50, 200, 500, 1000) on edgeR sensitivity and sensitivity at 5% FDR.

Supplementary Table S6. Jaccard similarity indices between originally published datasets or after reanalysis using edgeR or Stouffer's method in *Arabidopsis* and maize. The same numbers of topmost imprinted genes were selected from the reanalyzed datasets.

Supplementary Table S7. Effect of minimal read coverage cut-off on edgeR sensitivity and specificity in simulations at 5% FDR.
